# Change in muscle volume after steroid therapy in patients with myositis assessed using cross-sectional computed tomography

**DOI:** 10.1186/s12891-018-2008-8

**Published:** 2018-03-27

**Authors:** Takashi Nawata, Makoto Kubo, Takafumi Nomura, Keiji Oishi, Kosaku Shiragami, Tadayoshi Ikegami, Munemasa Okada, Shigeki Kobayashi, Masafumi Yano

**Affiliations:** 1 Department of Medicine and Clinical Science, Yamaguchi University Graduate School of Medicine, 1-1-1 Minamikogushi, Ube, Yamaguchi 755-8505 Japan; 2 Department of Radiology, Yamaguchi University Graduate School of Medicine, 1-1-1 Minamikogushi, Ube, 755-8505 Japan; 3 Department of Internal Medicine, Saiseikai Yamaguchi General Hospital, 2-11, Midorimachi, Yamaguchi, 753-8517 Japan

**Keywords:** Computed tomography, Cross section, Steroid, Muscle mass, Myositis

## Abstract

**Background:**

Steroid therapy, a key therapy for inflammatory, allergic, and immunological disorders, is often associated with steroid myopathy as one of the side effects. Steroid therapy is considered the first-line therapy for myositis; however, there have been no reports strictly comparing the muscle mass in patients with myositis before and after steroid therapy. Thus, it is currently unclear whether steroid therapy for such patients affects muscle volume in addition to muscle strength. We aimed to determine the change in muscle mass after steroid therapy via cross-sectional computed tomography (CT) in patients with myositis.

**Methods:**

Data from seven patients with myositis and eight controls, who were all treated with high doses of steroids, were assessed before and after steroid therapy. Clinical factors in patients with myositis included serum muscle enzyme levels and muscular strength. The cross-sectional area of skeletal muscle and the low muscle attenuation rate at the level of the caudal end of the third lumbar vertebra were obtained using CT and measured using an image analysis program for all patients. Data were subjected to statistical analysis using several well-established statistical tests. The Wilcoxon signed-rank test was used for comparing paired data for each patient. The Mann-Whitney U test was used to compare sets of data sampled from two groups. The Spearman’s rank correlation coefficient was used for determining the correlations between two variables. Statistical significance was set at *p* < 0.05.

**Results:**

Muscular strength and serum muscle enzyme levels improved following steroid therapy in patients with myositis. In both groups, the cross-sectional areas of skeletal muscles decreased (myositis group: *p* = 0.0156; control group: *p* = 0.0391) and the low muscle attenuation rate tended to increase (myositis group: *p* = 0.0781; control group: *p* = 0.0547). In the myositis group, patients with chronic obstructive pulmonary disease showed a tendency toward muscle volume loss (*p* = 0.0571).

**Conclusion:**

In patients with myositis treated with steroid therapy, muscle mass decreased after steroid therapy suggesting that the improvement in muscle strength was due to factors other than a change in muscle volume. Our study suggests the importance of therapies that not only improve muscle mass but also improve the quality of muscle strength.

## Background

Steroid therapy is a crucial form of treatment for inflammatory, allergic, and immunological disorders and has been used for > 50 years. Although steroid therapy is useful, there are various side effects, such as an associated increased susceptibility to infection or glucose intolerance [1].

Steroid myopathy is another side effect of steroid therapy, which is induced by the catabolic action of steroids on skeletal muscles [1, 2]. Muscle weakness in steroid myopathy usually begins in the proximal portion of the lower extremities, progresses to the upper proximal extremities, and finally affects the distal extremities [3]. It is known that steroid myopathy occurs in a dose-related manner. It has been reported that muscle strength in patients treated with > 40 mg/day of prednisone was significantly less than that in patients treated with < 40 mg/day [4].

Idiopathic inflammatory myopathies, collectively termed myositis, are autoimmune diseases characterized by skeletal muscle inflammation. Patients with myositis present with muscle weakness and atrophy in the proximal muscles and increases in serum levels of muscular enzymes [5]. Steroid therapy is considered the first-line therapy for myositis. With steroid therapy, patients with myositis show an improvement in muscle strength [6]; however, to our knowledge, there have been no reports strictly comparing the muscle mass of patients with myositis before and after steroid therapy. Therefore, it is unclear whether steroid therapy for patients with myositis improves not only muscle strength but also muscle volume.

Recently, several methods for quantifying muscle mass have been proposed. Bioelectrical impedance analysis and dual X-ray absorptiometry estimate the fat-free mass of the whole body or body segments [7, 8]. Computed tomography (CT) and magnetic resonance imaging (MRI) are also used to measure cross-sectional images that enable the estimation of muscle mass [9, 10]. Yoshizumi et al. reported that the cross-sectional area of skeletal muscle at the caudal end of the third lumbar vertebra, measured using CT, strongly correlated with body surface area [10]. It has been reported that MRI is a useful tool to diagnose muscle disorders. MRI makes it easy to assess not only muscle mass but also muscle quality, especially chemical shift imaging and Dixon based T2W imaging, which are new useful tools to assess fatty infiltration [11]. It has also been proposed that CT allows for the assessment of low attenuation of skeletal muscle (i.e., muscle with increased lipid content) [12]. With regard to the muscle volume of patients with rheumatic diseases, Hosono et al. reported that CT or MRI can estimate the steroid-related skeletal muscle loss in patients with rheumatic diseases more accurately than bioelectrical impedance analysis [13].

In this study, we retrospectively compared the changes in muscle volume before and after steroid therapy in patients with myositis and in steroid-treated patients without myositis by measuring the skeletal muscle area with CT.

## Methods

### Patients

Seven patients with recent-onset myositis (diagnosed from 2015 to 2017) were included in this study (five patients had dermatomyositis and two had mixed connective tissue disease). All patients fulfilled the diagnostic criteria proposed by Bohan and Peter [14]. The mean age was 55 years (range, 15–95 years) and four patients were female. The myositis group underwent high-dose steroid therapy (maximum dose of corticosteroid was > 0.7 mg/kg/day of prednisolone) (Table 1).

**Table 1:** Profiles of the patients in the myositis group

Patient	Age (years)	Sex	Diagnosis	Max glucocorticoid (mg/day)^a^	Duration between the start of treatment and second CT (months)^b^	Dose of glucocorticoid at second CT (mg/day)	Cumulative doses of glucocorticoid (g)^c^	MMT (before → after)^d^	Serum level of CK (U/L) (before → after)	Daily intake of protein in hospital (g/day)
1	26	M	DM	mPSL → PSL: 60	3	PSL: 25	PSL: 4.3	3+/3+ → 4+/4	102 → 21	80.0
2	58	F	DM	PSL: 40	2	PSL: 20	PSL: 1.7	4−/4- → 4/4-	500 → 17	54.0
3	74	F	DM	PSL: 45	2.5	PSL: 18	PSL: 2.3	4−/3+ → 4/4	3677 → 55	67.5
4	70	M	DM	mPSL → sPSL: 100	4	PSL: 22.5	PSL: 6.1	3−/3- → 4+/4+	5149 → 22	65.0
5	70	M	DM	PSL: 60	7.5	PSL: 12	PSL: 6.0	4+/4 → 5/5	1119 → 122	75.0
6	15	F	MCTD	PSL: 50	1	PSL: 50	PSL: 1.4	5−/5 → 5/5	876 → 219	70.0
7	69	F	MCTD	PSL: 40	3	PSL: 20	PSL: 2.7	4/4- → 5−/5-	2210 → 69	65.0

*M* male, *F* female, *DM* dermatomyositis, *MCTD* mixed connective tissue disease, *PSL* prednisolone, *mPSL* methylprednisolone, *sPSL* soluble prednisolone, *MMT* manual muscle test, creatine kinase

^a^Initial mPSL dose was 1 g × 3 d

^b^The first CTs were performed within six weeks before the initial steroid therapy

^c^Steroid taken using steroid pulse was excluded from cumulative doses of glucocorticoid

^d^Muscle strength of the bilateral iliopsoas muscle was evaluated using MMT before and after steroid therapy

Eight patients without myositis were also included as a control group. Of these patients, two had lupus nephritis, two had microscopic polyangiitis, two had minimal change nephrotic syndrome, one had eosinophilic granulomatosis with polyangiitis, and one had Henoch-Schönlein purpura nephritis. The mean age of the patients in the control group was 70 years (range, 46–94 years) and six patients were female. The control group also underwent high-dose steroid therapy at the same maximum corticosteroid dose as the myositis group. Patients who experienced disuse syndrome were excluded prior to the inclusion of the final eight patients in the control group (Table 2).

**Table 2:** Profiles of the patients in the control group

Patient	Age (years)	Sex	Diagnosis	Max glucocorticoid (mg/day)^a^	Duration between the start of treatment and second CT (months)^b^	Dose of glucocorticoid at second CT (mg/day)	Cumulative doses of glucocorticoid (g)^c,d^
1	70	M	MCNS	PSL: 40	1.5	PSL: 25	PSL: 1.4
2	66	F	LN	mPSL → PSL: 35	2.5	PSL: 20	PSL: 2.4
3	46	F	LN	mPSL → mPSL: 32	3	mPSL: 12	PSL: 2.2
4	57	F	EPGA	mPSL → PSL: 50	3	PSL: 25	PSL: 3.4
5	80	M	MPA	mPSL → PSL: 30	2.5	PSL: 15	PSL: 1.3
6	86	F	MPA	mPSL → mPSL: 32	1.5	mPSL: 20	PSL: 1.5
7	79	F	MCNS	mPSL: 32	1.5	mPSL: 16 + PSL: 2	PSL: 1.6
8	76	F	HSPN	mPSL: 32	5.0	mPSL: 8	PSL: 2.6

*M* male, *F* female, *MCNS* minimal change nephrotic syndrome, *LN* lupus nephritis, *EPGA* eosinophilic granulomatosis with polyangiitis, *MPA* microscopic polyangiitis, *HSPN* Henoch-Schönlein purpura nephritis, *PSL* prednisolone, *mPSL* methylprednisolone

^a^Initial mPSL dose was 500 mg × 3 d, except for Patient 3 (1 g × 3 d) and Patients 7 and 8 (reported in table)

^b^The first CTs were performed within 6 weeks before the initial steroid therapy

^c^Steroid taken using steroid pulse was excluded from cumulative doses of glucocorticoid

^d^mPSL 0.8 mg is converted as PSL 1 mg

### Assessment of muscular strength

The muscular strength of patients with myositis were measured using a manual muscle test (MMT) before and after steroid therapy.

### Computed tomography

**Fig. 1:**
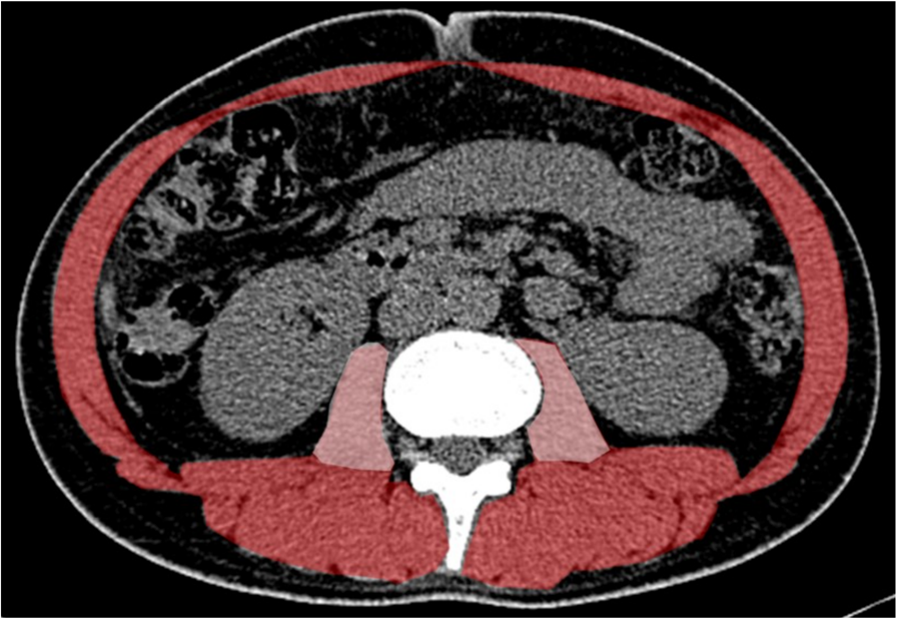
Measurement of the cross-sectional area of skeletal muscle at the level of the caudal end of the third lumbar vertebra. Muscle volume was measured in the psoas muscle and other muscles

All patients underwent CT to assess the general involvement before and after steroid therapy, and we retrospectively analyzed the change in muscle mass by comparing the CT findings. The first CT was performed within 6 weeks before the initiation of high-dose steroid therapy. The second CT was performed during the tapering of steroid therapy. CT data, including images of the caudal end of the third lumbar vertebra, were obtained using standard procedures. As indicated by a previous study, we defined values between − 29 and + 150 Hounsfield units (HU) as the skeletal muscle area (Fig. 1). Values between − 29 and + 30 HU were defined as low muscle attenuation [12]. Two radiologists measured the cross-sectional area of the skeletal muscles and the low muscle attenuation rate at the level of caudal end of the third lumbar vertebra. The mean attenuation of the muscles were measured using a semi-automatic image processing program, called “Image J”, that was written in Java, which allows the program to be run on Linux, Mac OS X, and Windows (both 32-bit and 64-bit modes) without a license (http://rsb.info.nih.gov/ij/).

### Statistical analysis

The inter-reader agreement for measuring the mean attenuation of muscle from the CT image was calculated using Cohen’s kappa statistic. The strength of agreement between the two readers was interpreted as poor (< 0.20), fair (0.21–0.40), moderate (0.41–0.60), good (0.61–0.80), or excellent (0.81–0.99) [15]. The Wilcoxon signed-rank test was used to compare paired data for each patient. The Mann-Whitney U test was used to compare two sets of data sampled from the two groups. The Spearman’s rank correlation coefficient was used to assess correlations between two variables. Comparisons were considered statistically significant at *p* < 0.05.

## Results

The inter-reader agreement showed excellent correlation between the two readers, ranging from 0.89–0.99, for the mean attenuation of the muscle and the rate of low muscle attenuation.

Seven patients with myositis and eight controls were analyzed in this study. The muscle strength, measured using MMT, improved after steroid therapy in all patients with myositis (Table 1). In addition, the serum level of creatine kinase also improved after steroid therapy in all patients with myositis ([mean ± SD] before: 1948 ± 1855 U/L vs after: 75 ± 74 U/L, *p* = 0.0156, analyzed using the Wilcoxon signed-rank test.) (Table 1). The most frequent underlying diseases in patients with myositis were chronic obstructive pulmonary disease (COPD) and interstitial lung disease followed by cancer (e.g., lung carcinoma, rectal carcinoma, pharyngeal carcinoma). No patients with cancer underwent chemotherapy. All underlying diseases were detected within 2 months of the diagnosis of myositis. Conversely, chronic kidney disease was the most frequent underlying disease in the control group (Table 3).

**Table 3:** Incidence of underlying diseases by group

	Myositis (*n* = 7)	Control (*n* = 8)
COPD	4 (57%)	2 (25%)
ILD	4 (57%)	4 (50%)
CKD	1 (14%)	6 (75%)
Cancer	3 (43%)	0 (0%)

*COPD* chronic obstructive pulmonary disease, *ILD* interstitial lung disease, *CKD* chronic kidney disease

**Fig. 2:**
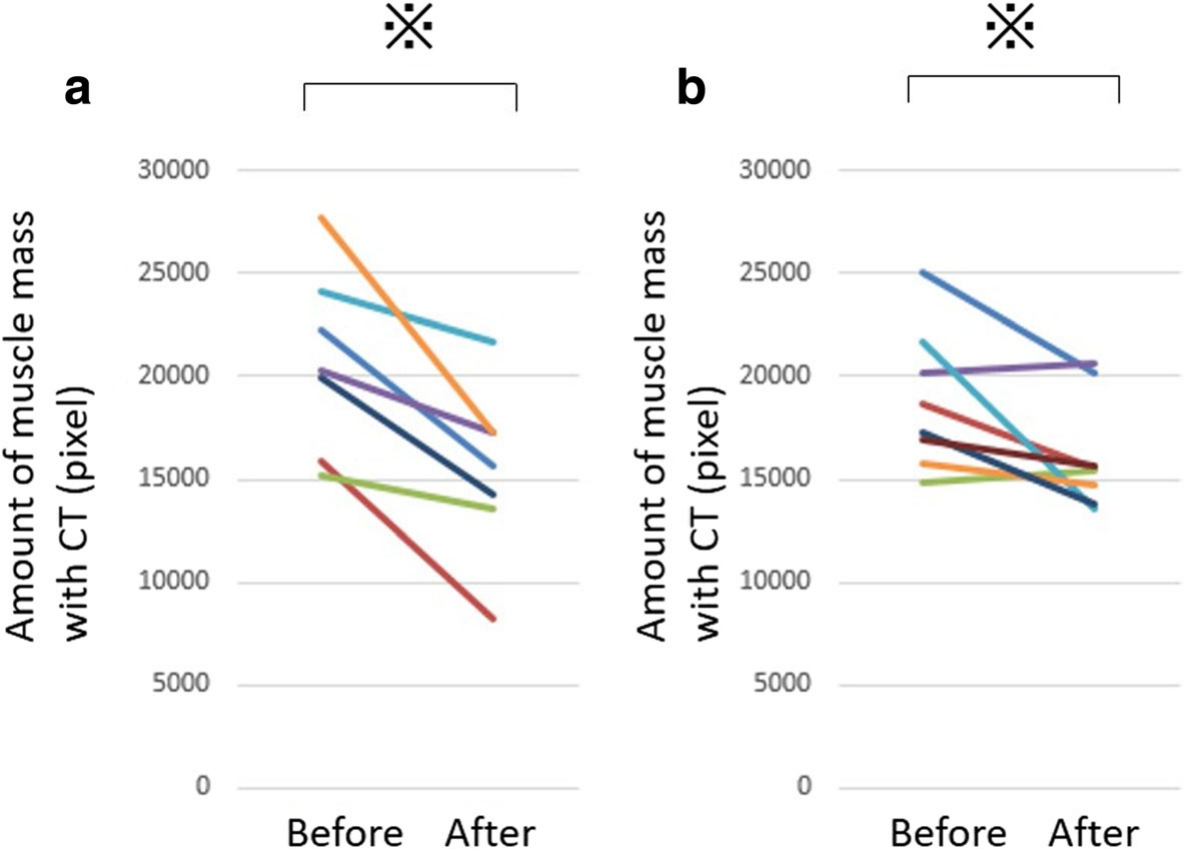
The muscle mass of both groups significantly decreased after steroid therapy: **a** myositis group: *p* = 0.0156 and **b** control group: *p* = 0.0391. *p < 0.05, analyzed using the Wilcoxon signed-rank test. *CT* computed tomography

**Fig. 3:**
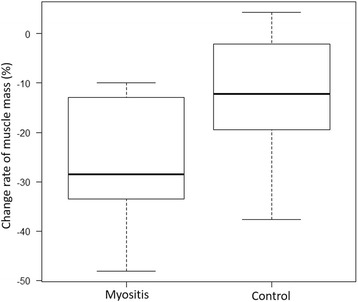
Though the myositis group showed greater muscle volume loss in comparison to the control group, the difference was not statistically significant (*p* = 0.121); analyzed using the Mann-Whitney U test

**Fig. 4:**
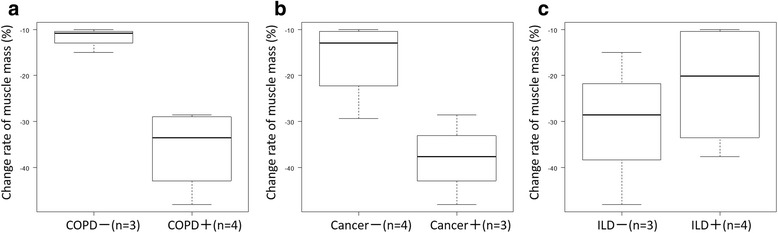
In the myositis group, patients with chronic obstructive pulmonary disease (COPD) showed a tendency toward muscle volume loss: **a** COPD: *p* = 0.0571; **b** Cancer: *p* = 0.114; **c** interstitial lung disease (ILD): *p* = 0.629, analyzed using the Mann-Whitney U test

**Fig. 5:**
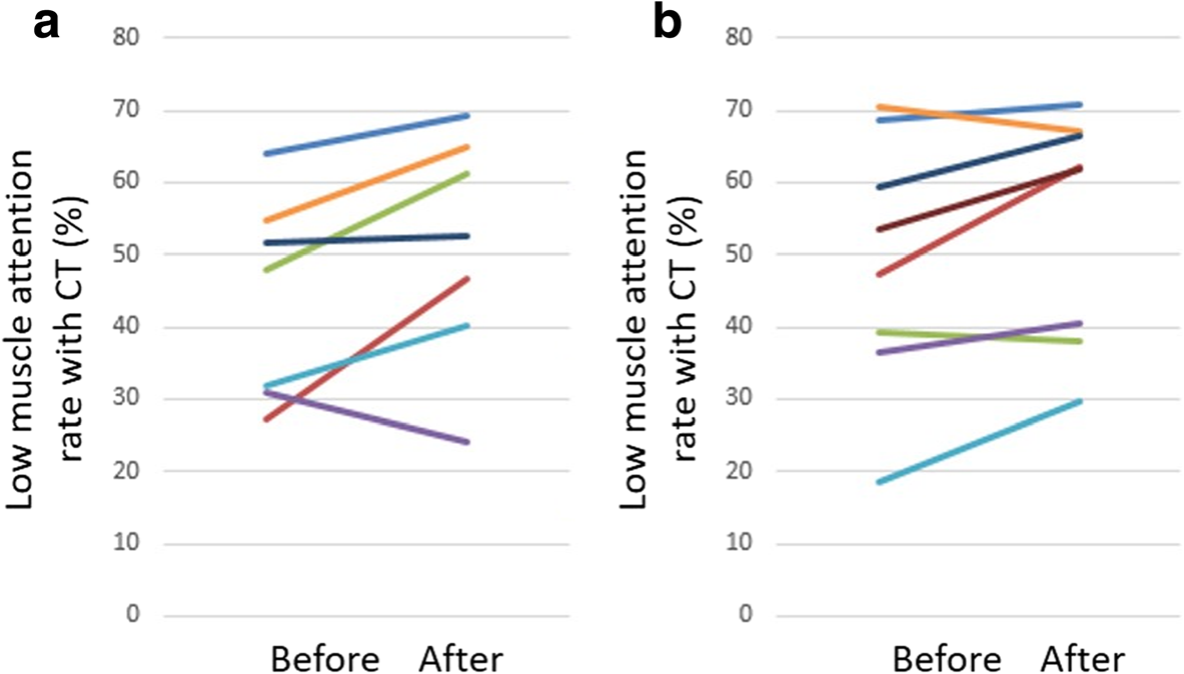
Both groups showed a tendency toward an increased low muscle attenuation rate after steroid therapy: **a** myositis group: *p* = 0.0781 and **b** control group: *p* = 0.0547, analyzed using the Wilcoxon signed-rank test. *CT* computed tomography

Muscle mass at the level of the caudal end of the third lumbar vertebra significantly decreased in both groups after steroid therapy ([mean ± SD] myositis group: − 25.6 ± 14.4%, control group: − 12.6 ± 14.6%) (Fig. 2). With regard to the rate of muscle volume change between the groups, although the myositis group showed greater muscle volume loss in comparison to the control group, the difference was not statistically significant (Fig. 3). In the myositis group, patients with COPD also showed a tendency toward muscle volume loss ([mean ± SD] myositis with COPD: − 35.9 ± 9.1%, (myositis without COPD: − 11.9 ± 2.7%). Though the myositis group with cancer showed greater muscle volume loss in comparison to the control group, the difference was not statistically significant ([mean ± SD] myositis with cancer: − 38.1 ± 9.8%, myositis without cancer: − 16.3 ± 9.0%). (Figure 4) in addition, both groups showed a tendency towards an increased low muscle attenuation rate following steroid therapy ([mean ± SD] myositis group: + 7.3 ± 8.5%, control group: + 5.4 ± 6.6%) (Fig. 5). In patients with myositis, correlations between the rate of change of muscle volume and low muscle attenuation and clinical factors (age, serum level of creatine kinase before treatment, cumulative doses of steroid, time between first CT and second CT, time between the start of high-dose steroid and second CT, and daily intake of protein in hospital) analyzed using the Spearman’s rank correlation coefficient were not significant.

## Discussion

Although all patients with myositis showed improvements in muscle strength and serum muscle enzyme levels after steroid therapy, there was a significant loss of muscle volume. The loss of muscle mass is induced by both extrinsic causes and intrinsic causes. Extrinsic causes of focal muscle atrophy often developed after traumatic injury such as Morel-Lavallee syndrome which is a post-traumatic closed degloving injury [16]. Our patients did not have a history of trauma. Therefore, we think that extrinsic causes had little involvement in the change of muscle mass in our patients. With regard to intrinsic causes, previous studies have reported that patients with cancer, COPD, and chronic kidney disease exhibited a loss of muscle volume [17–19]. In the present study, some of the patients had these conditions as underlying diseases. Therefore, the involvement of these underlying diseases may be associated with the observed muscle volume loss in our patients (especially in patients with cancer and COPD in the myositis group). However, the patients with myositis who did not develop these underlying diseases also showed a loss of muscle volume. This suggests that steroid therapy itself decreases muscle volume in patients with myositis. Thus, an improvement in muscle strength in patients with myositis may be attributed to other factors and not to muscle volume change.

Some mechanisms have been proposed to explain the etiology of the loss of muscle volume. One of these mechanisms is an imbalance of protein synthesis and catabolism [20]. Steroid myopathy is induced by the hypercatabolism of skeletal muscle, which might have resulted in the loss of muscle volume in our patients. However, despite the loss of muscle mass, improvement in muscle strength was also observed in patients with myositis who were treated with steroids. As such, we hypothesize that the improvement in muscle strength is influenced by an improvement in mitochondrial function. Currently, a non-immune cell mediated mechanism in myositis has been proposed [21]. It was reported that the endoplasmic reticulum (ER) stress pathways are chronically activated in patients with myositis [21]. Additionally, the biopsy specimens from patients with myositis and mice models of myositis demonstrated an increase in levels of Grp78 (also known as HSPA5), which is an ER stress-related protein [22]. The biopsy specimens from patients with myositis also showed co-localization of MHC class I and the ER marker, calnexin [22]. These results suggest the possibility of the involvement of ER stress in the pathogenesis of myositis. Xiao et al. reported that serum HSPA5 levels significantly decreased after steroid therapy in steroid-responsive patients with myositis. In contrast, serum HSPA5 levels did not change in non-responders [23]. The results of the previous study suggest that steroid therapy reduces ER stress in steroid-responsive patients with myositis. ER stress is known to increase reactive oxygen species, which are closely associated with mitochondrial dysfunction, depressed force generation, and activation of muscle catabolic and autophagy pathways [21]. We speculate that steroid therapy improves muscle strength via the reduction of ER stress and reactive oxygen species followed by an improvement in mitochondrial function. Our study suggests that the improvement in muscle strength after steroid therapy is not influenced by an improvement in muscle volume, but rather by an improvement in other factors, such as amelioration of mitochondrial dysfunction.

Our study has a few limitations. First, this was a retrospective study and the number of patients was small. The non-significant change in the low muscle attenuation rate after steroid therapy and the non-significant change between the rate of change of muscle volume in patients with myositis associated with COPD and cancer may be partly due to the small sample size. Second, the regimens for steroid reduction and the timing of implementation of the second CT were different for each of the patients, which makes it difficult to accurately assess the correlations between muscular changes and clinical factors. Third, though patients who experienced disuse syndrome were excluded from the control group, exact scores from the MMT (muscular strength) of the control group were unknown, which makes it difficult to assess the change of muscle strength in the control group. However, our study suggests the importance of therapies that not only improve muscle mass but also improve the quality of muscle strength for the treatment of myositis. Hence, this study may provide novel insights into therapies for myositis. Nonetheless, further studies are needed to reveal the mechanism and treatment of muscle weakness in patients with myositis.

## Conclusions

In patients with myositis treated with steroid therapy, muscle mass decreased after steroid therapy, suggesting that the improvement in muscle strength was due to factors other than changes in muscle volume.

## Abbreviations

CKD: Chronic kidney disease
COPD: Chronic obstructive pulmonary disease
CT: Computed tomography
EPGA: Eosinophilic granulomatosis with polyangiitis
ER: Endoplasmic reticulum
HSPN: Henoch-Schönlein purpura nephritis
ILD: Interstitial lung disease
LN: Lupus nephritis
MCSN: Minimal change nephrotic syndrome
MCTD: Mixed connective tissue disease
MMT: Manual muscle test
MPA: Microscopic Polyangiitis
mPSL: Methylprednisolone
MRI: Magnetic resonance imaging
PSL: Prednisolone
ROS: Reactive oxygen species
sPSL: Soluble prednisolone
